# Nasopharyngeal Angiofibroma in an Adult Male: A Case Report and Review of the Literature

**DOI:** 10.7759/cureus.49127

**Published:** 2023-11-20

**Authors:** Stephanie Choi, Vanessa J Zhang, Xiaoqin Zhu, Christopher J Ito

**Affiliations:** 1 Otolaryngology, UMass Chan Medical School, Worcester, USA; 2 Pathology, UMass Chan Medical School, Worcester, USA

**Keywords:** posterior nasal mass, tumor vascularity, nasal obstruction, epistaxis, endoscopic resection, nasopharyngeal angiofibroma

## Abstract

A 32-year-old male presented with recurrent bilateral epistaxis and nasal obstruction, leading to a rare diagnosis of nasopharyngeal angiofibroma. Although primarily observed in adolescents, this case underscores its presence in older populations. The patient underwent a successful endoscopic resection, contributing to the scant documented instances of such cases in adult U.S. males.

## Introduction

Nasopharyngeal angiofibroma, often termed “juvenile nasopharyngeal angiofibroma,” is a rare benign tumor characterized by its slow growth and pronounced vascularity [1]. It predominantly affects adolescent males aged 14 to 25, presenting typically with symptoms like epistaxis or nasal obstruction [1]. To date, only two cases have been reported in adult males within the United States [2,3]. The exact prevalence and incidence in adults are yet to be defined. Despite its benign nature, the tumor can pose significant morbidity due to its propensity for local invasiveness [4]. Complete surgical resection stands as the preferred treatment, with preoperative angiography aiding in visualizing tumor-related vessels and embolization [5]. In this context, we present a unique case of a 32-year-old male diagnosed with nasopharyngeal angiofibroma who successfully underwent an endoscopic resection of the tumor.

## Case presentation

A 32-year-old Hispanic male was evaluated at our otolaryngology clinic, presenting with recurrent bilateral epistaxis and escalating nasal obstruction that persisted over two years. During this period, he experienced several episodes of self-limiting bilateral epistaxis. He also had two severe instances of bilateral epistaxis managed with bilateral nasal packing. Concurrently, he described a series of severe headaches, exhibiting symptoms of phonophobia and photophobia, leading to a presumptive migraine diagnosis. His past medical history was otherwise unremarkable. 

**Figure 1:**
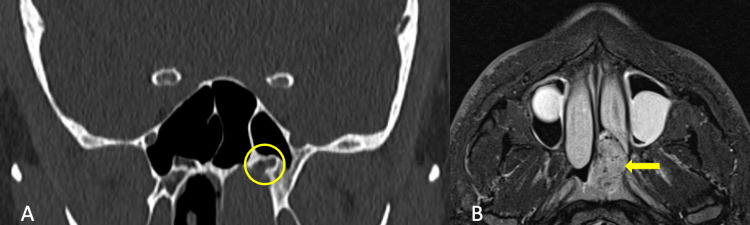
Radiological Findings of the Vascularized Mass in the Left Posterior Nasal Cavity (A) Computed tomography (CT) of the head revealing opacification in the left posterior nasal cavity and nasopharyngeal regions. The expanded vidian canal is highlighted with a circle. (B) Magnetic resonance imaging (MRI) of the face illustrating a 1.6 x 2.5 x 3.1 cm T2 hyperintense, T1 hypointense lesion with avid enhancement and flow voids, consistent with a vascularized mass. The tumor is indicated with an arrow.

Upon nasal endoscopic examination, a vascularized, smooth mass was detected, predominately filling the left posterior nasal cavity and left nasopharynx. Computed tomography (CT) of the head showed opacification in the left posterior nasal cavity and nasopharyngeal regions, with notable involvement of the vidian canal (Figure 1A). Magnetic resonance imaging (MRI) of the face detailed a 1.6 x 2.5 x 3.1 T2 hyperintense, T1 hypointense, avidly enhancing mass lesion with flow voids (Figure 1B). 

**Figure 2:**
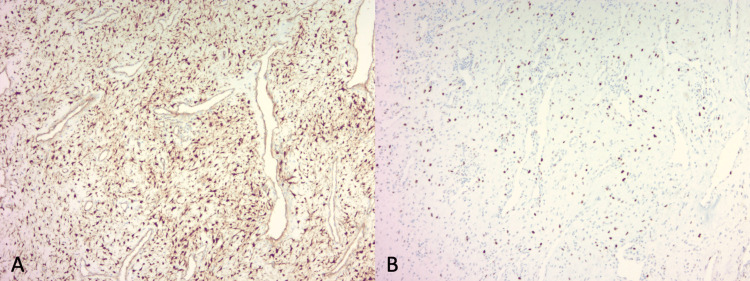
Immunohistochemical Findings of the Resected Nasal Mass Immunohistochemical staining of the excised lesion displaying diagnostic markers for nasopharyngeal angiofibroma. Both the beta-catenin and the androgen receptors are stained brown, indicating positive results. (A) Beta-catenin stain at 100x magnification. (B) Androgen receptor stain at 100x magnification.

**Figure 3:**
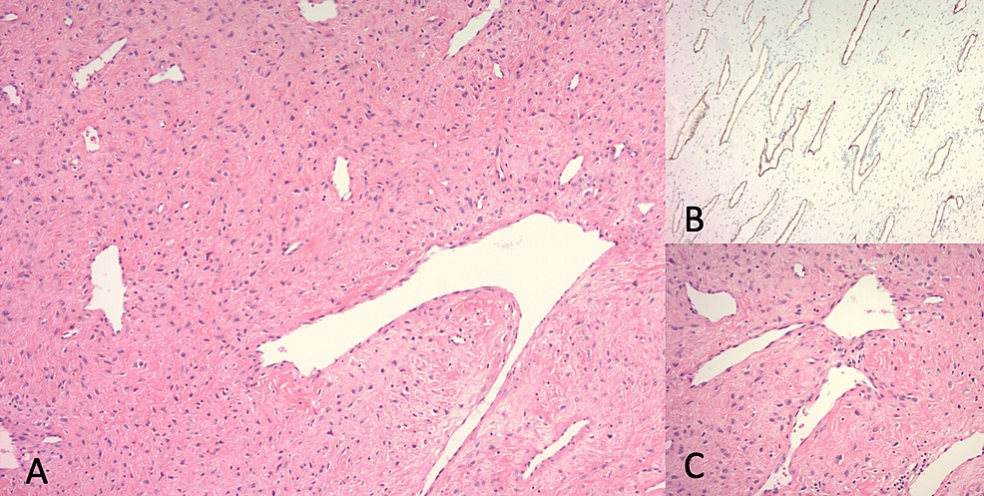
Vascular and Morphological Features of the Resected Nasal Mass Figure 3. Histopathological findings from the excised lesion illustrating the vascular nature and morphology of the nasopharyngeal angiofibroma. (A) Image at 100x magnification showing irregularly dilated vessels. (B) CD34 stain at 100x magnification highlighting the extensive vasculature of the tumor. (C) Image at 200x magnification focusing on the distinctive morphology of the stromal cells present within the tumor.

Two weeks following presentation, the patient underwent an endoscopic resection of the nasal mass. Preoperative angiography and embolization were not performed. The vascular mass extended from the left sphenoid face into the vidian canal and pterygoid fossa. A complete en-bloc resection with margins was performed, with the identification and ligation of the left internal maxillary artery as the primary feeding vessel. Intraoperative biopsy identified the mass as a mesenchymal spindle cell lesion, and post-operative histopathological examination of the excised lesion confirmed it as a nasopharyngeal angiofibroma. The specimen was notably vascular, with a stroma of stellate fibroblastic cells. Immunohistochemical staining of the stromal cells yielded positive results for beta-catenin and androgen receptors (Figure 2). The overall morphology and vascular characteristics of the tumor can be appreciated in Figure 3. The resected margins were negative. The patient had an uncomplicated postoperative course with good recovery. Follow-up nasal endoscopic examination and debridement revealed no residual tumor. The patient is scheduled for a six-month postoperative surveillance scan.

## Discussion

There is increasing evidence of adult cases of nasopharyngeal angiofibroma. A total of six case reports of histology-confirmed nasopharyngeal angiofibroma in adult males have been reported worldwide, with a noticeable bimodal age distribution in the documented reports (Table 1) [2-4,6-8]. In these reported cases, older patients tend to have more aggressive disease with extensive local invasion, whereas younger patients had more localized and less invasive disease [2-4,6-8]. Endoscopic resection of the nasopharyngeal angiofibroma was performed in all reported cases, with the majority of the cases reporting no evidence of disease at follow-up [2-4,6-8]. This study reports a rare case of nasopharyngeal angiofibroma in a 32-year-old adult male, strengthening the assertion that the term “juvenile nasopharyngeal angiofibroma” might indeed be a misnomer. Of note, our case is the third reported case of nasopharyngeal angiofibroma in adult males in the United States [2,3]. It is crucial for clinicians to consider this diagnosis in patients of all age groups, especially those presenting with recurrent epistaxis and posterior nasal masses. Further study is warranted to explore the differences in disease manifestation, pathophysiology, and risk factors of nasopharyngeal angiofibroma across different age groups.

**Table 1:** Characteristics, Imaging, Treatment, and Outcome of Adult Males with Histology-Confirmed Nasopharyngeal Angiofibroma CT, computed tomography; MRI, magnetic resonance imaging; NED, no evidence of disease.

Case report	Age/sex/race	Imaging findings	Treatment modality	Outcome
Stubbs et al. (2019) [2]	32/M/unknown	MRI demonstrated tumor extension into pterygopalatine fossa and cavernous sinus involvement	Bilateral medial maxillectomy, sphenoethmoidectomy, frontal sinusotomy, right middle turbinectomy, and posterior septectomy	Residual tumor at three months
Shah et al. (2019) [6]	32/M/unknown	CT demonstrated tumor localization in the right nasal cavity anteriorly	Intranasal endoscopic resection of tumor	NED at six months
McGarey et al. (2018) [3]	32/M/unknown	CT demonstrated broadening of the right pterygopalatine fossa with mass filling nasopharynx	Revision endoscopic sinus surgery	NED at six months
Sarafoleanu et al. (2011) [7]	56/M/unknown	CT and carotid angiography demonstrated a hypervascular tumor in the nasal cavity and osteolysis of the posterior part of the nasal septum, with the main blood supply from the internal maxillary artery	Lateral rhinotomy	NED at three years
Rahmadiyanto et al. (2022) [4]	62/M/Indonesian	CT demonstrated a solid lesion involving all paranasal sinuses with diffuse bony erosion and extension into the masticator space, nasopharyngeal space, retropharyngeal space, and temporalis muscles bilaterally. Note: medical history significant for bilateral recurrent NA for 15 years status post 12 endoscopic mass operations	Multidisciplinary surgical approach with otolaryngology, ophthalmology, neurosurgery, and plastic surgery	unknown
Zhang et al. (2015) [8]	72/M/Chinese	CT and MRI demonstrated an aggressive hypervascular mass centered in the right nasal cavity with extension into the maxillary sinus, masticator space, nasopharynx, and orbit, with perineural intracranial extension into the right middle cranial fossa and cavernous sinus	Endoscopy-assisted sublabial and buccolabial approach with preoperative embolization and incomplete resection followed by subsequent resection	NED at six months

## Conclusions

While nasopharyngeal angiofibroma is traditionally associated with adolescents, our case underscores the importance of considering this diagnosis across a broader age spectrum. Recognizing the presentations of nasopharyngeal angiofibroma in adults is vital for accurate diagnosis and effective management. Surgical resection remains the primary therapeutic approach, with a need for ongoing research to elucidate any distinct disease characteristics and management strategies for older patients.
